# Protein kinase a regulates cyclooxygenase-2 expression through the RNA-binding proteins HuR and TTP

**DOI:** 10.1016/j.jbc.2025.111064

**Published:** 2025-12-18

**Authors:** Sendi Rafael Adame-Garcia, Thomas S. Hoang, Pham Thuy Tien Vo, Valeria Burghi, Rodolfo Daniel Cervantes-Villagrana, Lennis Beatriz Orduña-Castillo, Dana J. Ramms, JoAnn Trejo, J. Silvio Gutkind

**Affiliations:** 1 Moores Cancer Center, University of California San Diego, La Jolla, California, USA; 2 Department of Pharmacology, School of Medicine, University of California San Diego, La Jolla, California, USA

**Keywords:** signal transduction, protein kinase A, RNA binding proteins, ciclooxigenase-2, postranscriptional regulation

## Abstract

Cyclooxygenase-2 (COX-2/PTGS2) is an inducible enzyme central to inflammatory responses, and its expression is tightly regulated. Elevated intracellular cAMP levels are known to stimulate COX-2 expression. However, the precise mechanism by which protein kinase A (PKA), the primary cAMP effector, mediates this process remains elusive. In this study, we investigated the role of PKA in regulating COX-2 expression in macrophages. We found that PKA activity is essential for COX-2 expression, primarily through a posttranscriptional mechanism that enhances COX-2 mRNA stability. This effect is mediated by the interaction between PKA and the RNA-binding proteins HuR (ELAVL1) and TTP (tristetraprolin/ZFP36). Specifically, we observed that the catalytic subunit of PKA directly interacts with HuR, Hu antigen R (HuR), a well-established COX-2 mRNA stabilizer. PKA activation increased HuR binding to COX-2 mRNA, and pharmacological inhibition of HuR abrogated COX-2 expression in macrophages stimulated with PGE_2_ and interleukin-1β. Furthermore, PKA stimulates the phosphorylation of TTP, an mRNA-destabilizing protein, thereby reducing its binding to the COX-2 transcript. We propose that PKA enhances COX-2 expression by interacting with HuR, maintaining proximity to COX-2 mRNA, and protecting it from TTP-mediated destabilization. Our findings reveal a mechanistic link between PKA activity and COX-2 mRNA stability through HuR and TTP, highlighting the role of RNA-binding proteins as novel effectors of PKA signaling in posttranscriptional regulation.

Cyclooxygenase-2 (COX-2) plays a central role in inflammation and immune responses by catalyzing the committed step for prostaglandin (PG) synthesis, including PGE_2_ (1). Unlike its constitutively expressed isoform COX-1, COX-2 expression is inducible, transient, and tightly regulated at multiple levels (1, 2, 3). Regulation of COX-2 mRNA expression involves the activation of NF-κB, c-Jun/c-Fos (AP-1), and C/EBPβ (4, 5, 6). Posttranscriptional mechanisms include the targeting of multiple AU-rich elements (AREs) at 3′ untranslated region (UTR) by RNA-binding proteins (RBPs) like HuR/ELAVL1 (Human antigen R/ELAV-like protein 1) and TTP/ZFP36 (Tristetraprolin), which modulate COX-2 mRNA stability (7, 8, 9, 10). In addition, posttranslational modifications (PTMs) such as *N*-glycosylation and ubiquitination, as well as substrate-dependent suicide inactivation, control COX-2 protein degradation (11, 12, 13).

Various environmental stimuli, including bacterial lipopolysaccharide (LPS), ceramides, and proinflammatory interleukins (ILs), as well as chemotherapeutic drugs, induce COX-2 expression by activating signaling pathways that converge toward transcription factors such as NF-κB and AP-1 (4, 5, 6). Elevated intracellular cAMP levels, triggered by Gs-coupled receptors (Gs-GPCRs) such as EP2 and EP4 for PGE_2_, also stimulate COX-2 expression, which establishes a positive feedback loop that sustains PGE_2_ production (14, 15, 16, 17, 18).

Previous studies have described multiple mechanisms for cAMP-mediated COX-2 induction, including CREB and AP-1 transcriptional activity at cAMP response elements (CRE) and p38^MAPK^-dependent mRNA stabilization (14). However, the direct role of protein kinase A (PKA), the primary cAMP effector, in these processes remains poorly understood. In this study, we aimed to investigate the direct involvement of PKA in COX-2 induction in macrophages. We identified a novel mechanism whereby PKA regulates COX-2 expression through the modulation of two RBPs: Hu antigen R (HuR) and TTP. Specifically, we found that PKA interacts with HuR, a COX-2 mRNA stabilizer, and phosphorylates and inhibits TTP, a COX-2 mRNA destabilizer. We propose that PKA interaction with HuR enhances its stabilizing activity, maintaining PKA in proximity to the COX-2 transcript, protecting it from TTP-mediated destabilization through direct phosphorylation at serine 273.

## Results

### PKA activity is required for the induction and stabilization of COX-2 mRNA

**Figure 1:**
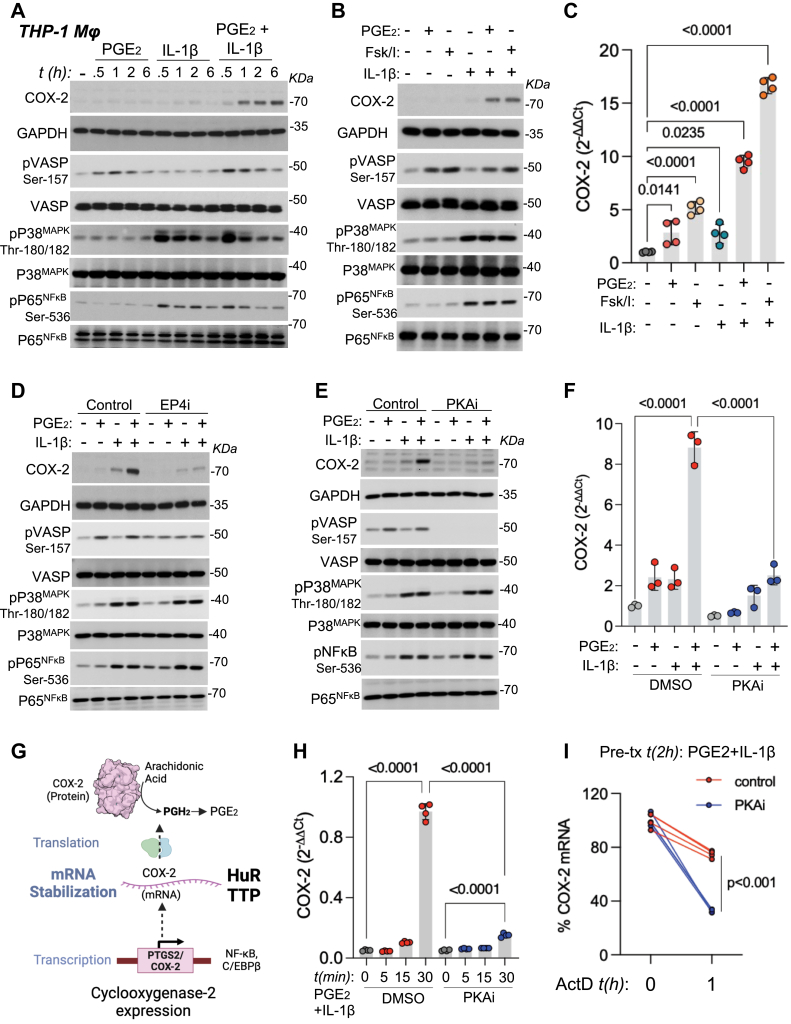
**PKA activation is required for COX-2 induction and stabilization in macrophages**. *A*, THP-1 macrophages (Mϕ) were serum-starved for 12 h and stimulated with 1 μM PGE_2_, 5 ng/ml IL-1β, or a combination of both for the indicated times. Cell lysates were analyzed by immunoblotting for COX-2 and GAPDH. Representative blots from three independent experiments are shown. *B*, THP-1 Mϕ were serum-starved for 12 h and stimulated with 1 μM PGE_2_, 1 μM forskolin/50 μM IBMX, 5 ng/ml IL-1β, or combinations for 6 h. Cell lysates were analyzed by immunoblotting for COX-2 and GAPDH. Representative blots from three independent experiments are shown. *C*, THP-1 Mϕ were serum-starved for 12 h and stimulated as in *B* for 6 h. Total RNA was extracted, and COX-2 and GAPDH mRNA levels were determined by RT-qPCR. Graph shows ΔΔCt values (mean ± SD) from four independent experiments (n = 4). Statistical significance was determined by one-way ANOVA followed by Sidak's multiple comparisons test; *p* values are indicated. *D*, THP-1 Mϕ were serum-starved for 12 h, preincubated with 1 μM ONO-AE3-208 (EP4 inhibitor) for 1 h, and then stimulated with 1 μM PGE_2_, 5 ng/ml IL-1β, or a combination for 6 h. Cell lysates were analyzed by immunoblotting for COX-2 and GAPDH. Representative blots from three independent experiments are shown. *E*, THP-1 Mϕ were serum-starved for 12 h, preincubated with 1 μM BLU0588 (PKA inhibitor) for 12 h, and then stimulated with 1 μM PGE_2_, 5 ng/ml IL-1β, or a combination for 6 h. Cell lysates were analyzed by immunoblotting for COX-2 and GAPDH. Representative blots from three independent experiments are shown. *F*, THP-1 Mϕ were serum-starved for 12 h, preincubated with 1 μM BLU0588 for 12 h, and then stimulated as in *E* for 6 h. Total RNA was extracted, and COX-2 and GAPDH mRNA levels were determined by RT-qPCR. Graph shows ΔΔCt values (mean ± SD) from three independent experiments (n = 3). Statistical significance was determined by one-way ANOVA followed by Sidak's multiple comparisons test; *p* values are indicated. *G*, schematic representation of COX-2 regulation at transcriptional, posttranscriptional (RNA stability), and protein stability levels. *H*, THP-1 Mϕ were stimulated with 1 μM PGE_2_ and 5 ng/ml IL-1β for the indicated short time points. Total RNA was extracted, and COX-2 mRNA levels were determined by RT-qPCR. Graph shows ΔΔCt values (mean ± SD) from three independent experiments (n = 3). Statistical significance was determined by one-way ANOVA followed by Bonferroni's multiple comparisons test; *p* values are indicated. *I*, THP-1 Mϕ were serum-starved for 12 h, stimulated with 1 μM PGE_2_ and 5 ng/ml IL-1β for the indicated times followed by the addition of 5 μg/ml actinomycin D to inhibit transcription for the indicated time points, in the presence or absence of 1 μM PKA inhibitor BLU0588. Total RNA was extracted, and COX-2 mRNA levels were determined by RT-qPCR. Graph shows the percentage of COX-2 mRNA remaining at each time point, normalized to time 0, from three independent experiments. COX-2, cyclooxygenase-2; IL-1β, interleukin 1β; Mϕ, macrophages; PKA, protein kinase A; RT-qPCR, reverse transcription quantitative PCR.

Based on previous studies (19, 20), we stimulated THP-1 macrophages (Mϕ) with PGE_2_ and IL-1β as a cellular model system to investigate COX-2 expression. We observed a significant increase in COX-2 expression following combinatorial stimulation with PGE_2_ and IL-1β compared to individual treatments (Fig. 1*A*). This enhanced COX-2 expression was accompanied by the phosphorylation of mitogen-activated protein kinase (MAPK) p38, the nuclear factor kappa-light-chain-enhancer of activated B cells (NFκB) p65 subunit, and the PKA substrate vasodilator-stimulated phosphoprotein (VASP) (Fig. 1A). We hypothesized that PKA activation plays a critical role in COX-2 induction. To test this, we activated PKA by increasing intracellular cAMP levels using forskolin and IBMX in combination with IL-1β. This treatment recapitulated the effects of PGE_2_ on COX-2 protein and mRNA induction (Fig. 1, *B* and *C*), suggesting that PGE_2_ stimulates Gs-coupled EP receptors, leading to cAMP production and subsequent PKA activation, which contributes to increased COX-2 expression. We identified Gs-coupled EP4 as the major PGE_2_ receptor expressed in THP-1 macrophages (Fig. S1*A*). Blocking this receptor with the selective antagonist ONO-AE3-208 prevented COX-2 induction by PGE_2_ and IL-1β costimulation (Fig. 1*D*). Furthermore, confirming the role of PKA in COX-2 induction, the selective PKA inhibitor BLU0588 successfully prevented COX-2 protein and mRNA expression induced by PGE_2_ and IL-1β costimulation (Fig. 1, *E* and *F*). These results indicate that PKA activity downstream of EP4 stimulation is required for COX-2 induction by combinatorial treatment with PGE_2_ and IL-1β. Given that COX-2 expression is regulated at transcriptional, posttranscriptional, and posttranscriptional levels (Fig. 1*G*), we sought to determine the level at which PKA exerts its regulatory effect. We found that PKA inhibition by BLU0588 did not alter COX-2 protein decay following cycloheximide treatment (Fig. S1*B*). However, short-term COX-2 mRNA expression was significantly downregulated by PKA inhibition with BLU0588 (Fig. 1*H*), suggesting that PKA primarily regulates COX-2 at the mRNA level. Consistent with this, PKA inhibition accentuated the decrease in COX-2 mRNA levels in mRNA decay assays using Actinomycin D (Fig. 1*I*). Collectively, our findings suggest that PKA contributes to COX-2 induction through a mechanism that involves mRNA stabilization.

### PKA interacts with HuR, a regulator of COX-2 mRNA stability

**Figure 2:**
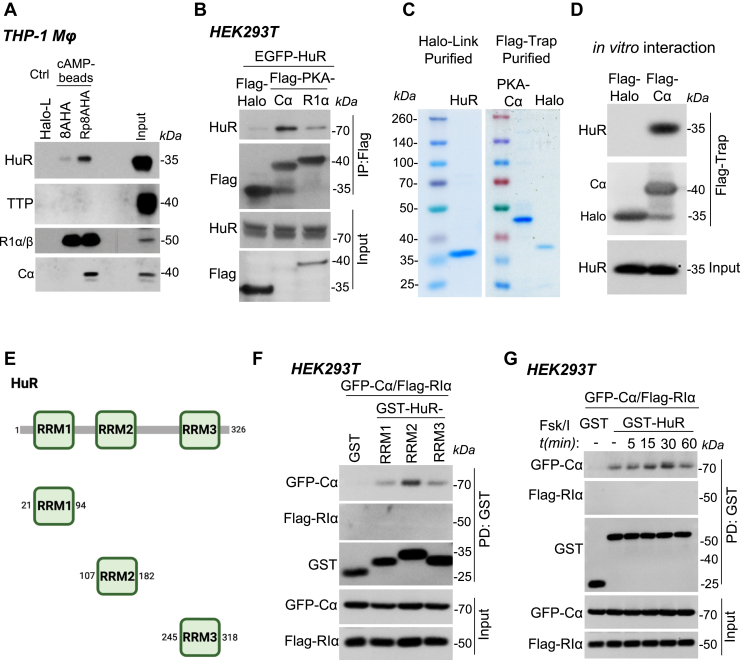
**PKA interacts with HuR, a regulator of COX-2 mRNA stability**. *A*, interaction of endogenous PKA with HuR in THP-1 macrophages (Mϕ). THP-1 Mϕ were serum-starved for 12 h and stimulated with 100 ng/ml LPS for 2 h. Cell lysates were subjected to pull-down using 8-AHA-cAMP (RIα), Rp-8-AHA-cAMPS (holoenzyme), or HaloLink resin (control), followed by immunoblot analysis for HuR, TTP, PKA-Cα, and RIα. *B*, interaction of HuR with the PKA catalytic subunit. HEK293T cells expressing EGFP-HuR, Flag-Cα, Flag-RIα, and Flag-HaloTag (negative control) were serum-starved for 12 h. Cell lysates were immunoprecipitated using DYKDDDDK Fab-Trap agarose and analyzed by immunoblotting for EGFP and Flag. *C*, purification of HuR and PKA-Cα for *in vitro* interaction assays. Coomassie blue-stained gels showing purified HaloTag-TEV-HuR (soluble) and DYKDDDDK Fab-Trap agarose-immobilized PKA-Cα and HaloTag (negative control). *D*, *in vitro* interaction between HuR and PKA-Cα. Purified PKA-Cα or HaloTag (immobilized on beads) was incubated with purified HuR (soluble). Bound HuR was detected by immunoblotting. *E*, schematic representation of HuR's RNA recognition motifs (RRMs) used to generate GST-tagged constructs for interaction studies. *F*, interaction of PKA-Cα with HuR RRM domains. HEK293T cells coexpressing GST-HuR RRM domains, GFP-PKA-Cα, and PKA-RIα were subjected to GST pull-down and analyzed by immunoblotting for GFP, Flag, and GST. *G*, enhancement of HuR-PKA-Cα interaction by forskolin/IBMX stimulation. HEK293T cells expressing GST-HuR, GFP-PKA-Cα, and PKA-RIα were serum-starved for 12 h and stimulated with 1 μM forskolin and 50 μM IBMX for the indicated times. Cell lysates were subjected to GST pull-down and analyzed by immunoblotting for GFP, Flag, and GST. COX-2, cyclooxygenase-2; HEK293T, human embryonic kidney 293 cell line; HuR, Hu antigen R; LPS, lipopolysaccharide; PKA, protein kinase A; TTP, tristetraprolin.

COX-2 mRNA stability is primarily balanced by the interplay of the RBPs, HuR and TTP, which recognize 22 AREs located in the 3′ UTR of COX-2 mRNA. TTP binding to COX-2 AREs initiates the recruitment of the deadenylating complex CCR4-NOT1, leading to poly(A) tail degradation and mRNA destabilization (9). Conversely, HuR competes with TTP for binding to COX-2 AREs, preventing the recruitment of degradation complexes (9, 21). We hypothesized that HuR and TTP could link PKA to COX-2 mRNA stabilization. To test this hypothesis, we used immobilized cAMP analogs to isolate either PKA regulatory subunits (8AHA-cAMP-agarose) or PKA holoenzyme (Rp-8AHA-cAMPS-agarose) from THP-1 macrophage lysates and detect potential associations with HuR or TTP (Fig. S2*A*). We did not detect an association with endogenous TTP in the pull-down assays (Fig. 2*A*). However, we found that endogenously expressed HuR coprecipitated efficiently with endogenous PKA holoenzyme (Fig. 2*A*) but was weakly associated with the endogenous RIα/β regulatory subunits, suggesting a requirement of PKA catalytic subunits (Cα) for the interaction. To assess this, we performed immunoprecipitation of Flag-tagged PKA-Cα or RIα subunits from human embryonic kidney 293T (HEK293T) cells transfected with the corresponding constructs. Consistent with the cAMP-agarose experiments, EGFP-tagged HuR preferentially associated with Flag-PKA-Cα and exhibited a weak interaction with RIα (Fig. 2*B*). To evaluate if this interaction is direct, we purified soluble HuR and Flag-Trap immobilized PKA-Cα proteins (Fig. 2*C*) and performed immunoprecipitation. We found that purified HuR coprecipitated with immobilized Cα (Fig. 2*D*), suggesting a direct protein-protein interaction. We then sought to determine the minimal region of HuR that interacts with PKA-Cα. We expressed individual RNA recognition motifs (RRMs) of HuR (Fig. 2*E*) tagged with GST in HEK293T cells and performed pull-down experiments. We found that PKA-Cα preferentially interacts with the second RRM domain of HuR (Fig. 2*F*), while maintaining a weak interaction with RRM1 and RRM3. Coprecipitation of Flag-RIα was not detected. The preferential interaction of HuR with the PKA catalytic subunit suggests that the interaction may be influenced by holoenzyme dissociation through increased cAMP levels. To evaluate this possibility, we performed pull-downs of HuR from transfected HEK293T cells stimulated with forskolin and IBMX at different time points. We observed that cAMP stimulation led to a transient increase in the association of GST-HuR with EGFP-PKA-Cα (Fig. 2*G*). Coprecipitation of Flag-RIα was not detected. To gain insights whether the interaction between PKA-Cα and HuR is sterically compatible with the functional activities of both proteins, protein kinase activity and RNA binding, respectively, we employed AlphaFold 3 to generate *in silico* structural models of the complex. First, we generated independent structural models for the PKA-Cα and the RRM1-2 domains of HuR. Visualization of these individual structures allowed us to map and highlight previously characterized functional sites, including the regulatory subunit binding, ATP-binding and the catalytic sites on PKA-Cα (Fig. S2*B*) and the RNA-binding sites on the HuR RRM domains (Fig. S2*C*). Subsequently, we generated 25 models for the PKA-Cα/HuR complex. The model with the highest interface predicted template modeling (ipTM) score suggest that the PKA primarily interacts with the second RRM domain of HuR (Fig. S2, *D* and *E*). This predicted interaction site is consistent with our experimental findings (Fig. 2*F*). This model also suggests that several functional regions remain sterically unoccupied in the PKA-Cα/HuR complex, including the catalytic and the regulatory subunit binding site on PKA and the RNA-binding site on the RRM1 domain of HuR. These findings suggest that the PKA-Cα/HuR complex could preserve the regulation and function of the PKA protein kinase and the RNA-binding capability of HuR. Together, our findings reveal a previously unidentified protein-protein interaction between the PKA catalytic subunit and the mRNA stabilizer HuR, which could potentially link PKA activity to COX-2 mRNA stabilization.

### HuR inhibition prevents PKA-mediated COX-2 expression

**Figure 3:**
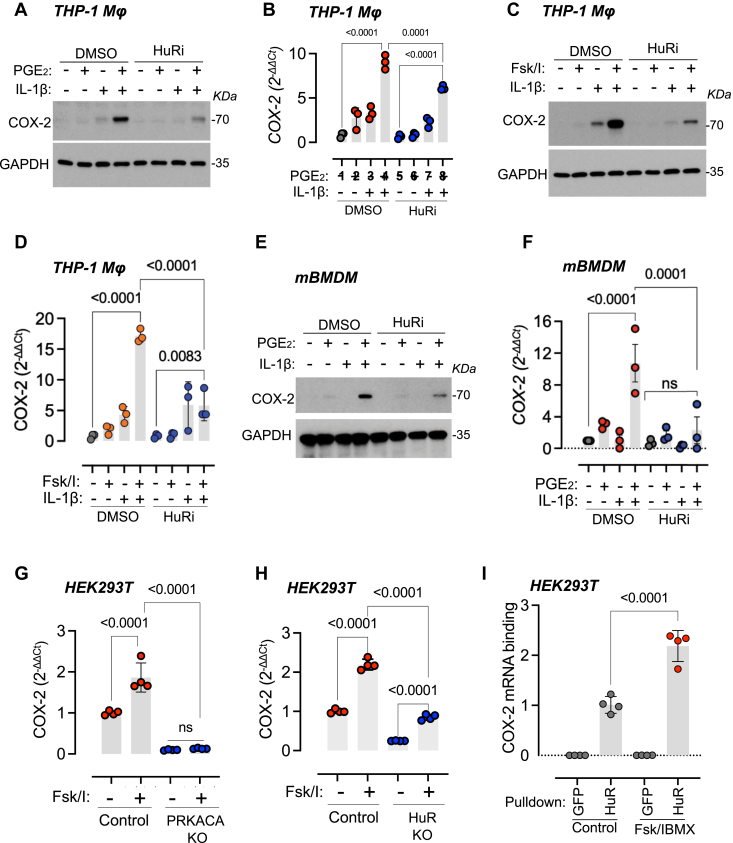
**HuR inhibition prevents COX-2 expression induced by PGE_2_ and IL-1β in macrophages**. *A*, effect of HuR inhibition on COX-2 protein expression in THP-1 macrophages (Mϕ) stimulated with PGE_2_ and IL-1β. THP-1 Mϕ were serum-starved for 12 h, preincubated with 1 μM MS-444 (HuR inhibitor) for 12 h, and then stimulated with 1 μM PGE_2_, 5 ng/ml IL-1β, or a combination for 6 h. Cell lysates were analyzed by immunoblotting for COX-2 and GAPDH. Representative blots from three independent experiments are shown. *B*, effect of HuR inhibition on COX-2 mRNA expression in THP-1 Mϕ stimulated with PGE_2_ and IL-1β. THP-1 MΦ were treated as in *A*. Total RNA was extracted, and COX-2 and GAPDH mRNA levels were determined by RT-qPCR. Graph shows ΔΔCt values (mean ± SD) from three independent experiments (n = 3). Statistical significance was determined by one-way ANOVA followed by Sidak's multiple comparisons test; *p* values are indicated. *C*, effect of HuR inhibition on COX-2 protein expression in murine bone marrow-derived macrophages (mBMDMs) stimulated with PGE_2_ and IL-1β. mBMDMs were treated as in *A*. Cell lysates were analyzed by immunoblotting for mCOX-2 and mGAPDH. Representative blots from three independent experiments are shown. *D*, effect of HuR inhibition on COX-2 mRNA expression in mBMDMs stimulated with PGE_2_ and IL-1β. mBMDMs were treated as in *A*. Total RNA was extracted, and COX-2 and GAPDH mRNA levels were determined by RT-qPCR. Graph shows ΔΔCt values (mean ± SD) from three independent experiments (n = 3). Statistical significance was determined by one-way ANOVA followed by Sidak's multiple comparisons test; *p* values are indicated. *E*, effect of HuR inhibition on COX-2 protein expression in THP-1 Mϕ stimulated with forskolin, IBMX, and IL-1β. THP-1 Mϕ were serum-starved for 12 h, preincubated with 1 μM MS-444 for 12 h, and then stimulated with 1 μM forskolin, 50 μM IBMX, and 5 ng/ml IL-1β, or a combination for 6 h. Cell lysates were analyzed by immunoblotting for COX-2 and GAPDH. Representative blots from three independent experiments are shown. *F*, effect of HuR inhibition on COX-2 mRNA expression in THP-1 Mϕ stimulated with forskolin, IBMX, and IL-1β. THP-1 Mϕ were treated as in *E*. Total RNA was extracted, and COX-2 and GAPDH mRNA levels were determined by RT-qPCR. Graph shows ΔΔCt values (mean ± SD) from three independent experiments (n = 3). Statistical significance was determined by one-way ANOVA followed by Sidak's multiple comparisons test; *p* values are indicated. *G*, effect of PKA-Cα knockout (KO) on COX-2 mRNA expression in HEK293T cells stimulated with forskolin and IBMX. HEK293T cells (Cas9 control or PKA-Cα KO) were serum-starved for 12 h and stimulated with 1 μM forskolin and 50 μM IBMX for 1 h. Total RNA was extracted, and COX-2 and GAPDH mRNA levels were determined by RT-qPCR. Graph shows ΔΔCt values (mean ± SD) from four independent experiments (n = 4). Statistical significance was determined by one-way ANOVA followed by Sidak's multiple comparisons test; *p* values are indicated. *H*, effect of HuR knockout (KO) on COX-2 mRNA expression in HEK293T cells stimulated with forskolin and IBMX. HEK293T cells (Cas9 control or HuR KO) were treated as in *G*. Graph shows ΔΔCt values (mean ± SD) from four independent experiments (n = 4). Statistical significance was determined by one-way ANOVA followed by Sidak's multiple comparisons test; *p* values are indicated. *I*, effect of PKA and HuR KO on COX-2 protein expression in HEK293T cells stimulated with forskolin and IBMX. HEK293T cells (Cas9 control, PKA-Cα KO, or HuR KO) were serum-starved for 12 h and stimulated with 1 μM forskolin and 50 μM IBMX for 1 h. Cell lysates were analyzed by immunoblotting for COX-2 and GAPDH. Representative blots from three independent experiments are shown. COX-2, cyclooxygenase-2; HuR, Hu antigen R; IL-1β, interleukin 1β; PKA, protein kinase A; RT-qPCR, reverse transcription quantitative PCR.

Given the direct interaction between PKA-Cα and HuR, we investigated whether this association is functionally linked to COX-2 stabilization downstream of PGE_2_ and IL-1β costimulation. To this end, we performed pharmacological inhibition of HuR using the small molecule MS-444. We observed that HuR inhibition decreased COX-2 protein and mRNA expression in THP-1 macrophages costimulated with PGE_2_ and IL-1β (Fig. 3, *A* and *B*) or forskolin/IBMX and IL-1β (Fig. 3, *C* and *D*). Furthermore, inhibition of HuR also decreased the PGE_2_ secretion from THP-1 macrophages costimulated with forskolin/IBMX and IL-1β (Fig. S2*F*). THP-1 cells are widely used as an experimental system for studying macrophage biology and signaling. However, these cells are derived from an acute myeloid leukemia patient and may not fully represent physiological conditions. As such, we decided to strengthen our studies by confirming our results in murine bone marrow-derived macrophages (mBMDMs). Similarly to the observations in THP-1 macrophages, COX-2 expression was markedly reduced by HuR inhibition in mBMDMs costimulated with PGE_2_ and IL-1β (Fig. 3, *E* and *F*). Consistent with these findings, PKA-Cα and HuR CRISPR-knockout prevented COX-2 mRNA (Fig. 3, *G* and *H*) and protein (Fig. S3, *A* and *B*) expression induced by forskolin and IBMX in HEK293T cells. Importantly, CREB phosphorylation (Fig. S3*C*) and transcriptional activity on CRE (Fig. S3*D*), measured by luciferase reporter assay, remained unaltered in HuR knockout cells, reinforcing the idea that HuR-dependent effects of PKA on COX-2 expression occur at the posttranscriptional level. Since HuR exerts its stabilizing effects through direct interaction with mRNA, we hypothesized that PKA activation could promote HuR binding to the COX-2 transcript. To evaluate this, we performed RNA immunoprecipitation of EGFP-HuR expressed in HEK293T cells stimulated with forskolin and IBMX, followed by quantitative PCR (qPCR) and end point PCR. We found that PKA stimulation increased HuR binding to COX-2 mRNA (Fig. 3, *I* and *E*). Collectively, our results suggest that PKA-mediated COX-2 induction involves HuR and its binding to COX-2 mRNA.

### PKA promotes the phosphorylation of TTP at serine 273, decreasing its binding to COX-2 mRNA

**Figure 4:**
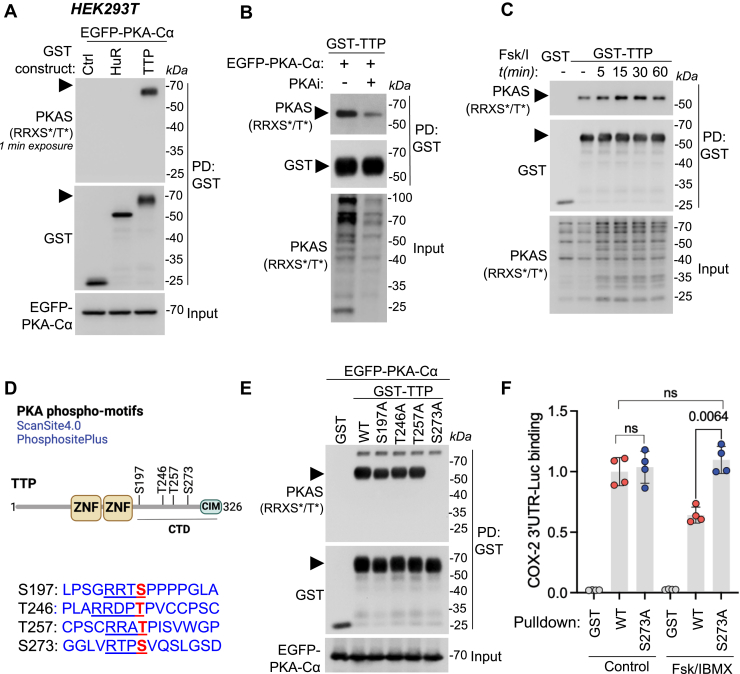
**PKA promotes phosphorylation of TTP at serine 273 and decreases its binding to the COX-2 3′UTR**. *A*, interaction of PKA with HuR or TTP. HEK293T cells coexpressing EGFP-PKA-Cα and GST-tagged HuR or TTP were lysed and subjected to GST pulldown. Pull-down and input samples were analyzed by immunoblotting for PKAS, GST, and GFP. *B*, effect of PKA inhibition on TTP interaction. HEK293T cells coexpressing EGFP-PKA-Cα and GST-tagged TTP were incubated with 1 μM BLU0588 (PKA inhibitor) for 12 h, lysed, and subjected to GST pulldown. Pulldown and input samples were analyzed by immunoblotting for PKAs and GST. *C*, effect of forskolin/IBMX stimulation on TTP interaction. HEK293T cells expressing GST-TTP were serum-starved for 12 h, stimulated with 1 μM forskolin and 50 μM IBMX for the indicated times, lysed, and subjected to GST pull-down. Pull-down and input samples were analyzed by immunoblotting for PKAs and GST. *D*, schematic representation of TTP modular architecture and potential PKA phosphorylation sites based on Scansite 4.0 and PhosphoSitePlus. *E*, identification of PKA phosphorylation site on TTP. HEK293T cells coexpressing EGFP-PKA-Cα and GST-tagged TTP wild-type or Ser/Thr mutants were lysed and subjected to GST pulldown. Pulldown and input samples were analyzed by immunoblotting for PKAs, GST, and GFP. Representative blots from three independent experiments are shown. *F*, effect of PKA activation on TTP interaction with COX-2 3′UTR. HEK293T cells coexpressing GST, GST-TTP wild-type or S273A, and COX-2 3′UTR-luciferase were serum-starved, treated with 1 μM forskolin and 50 μM IBMX for 1 h, lysed, and subjected to RNA immunoprecipitation (RIP). COX-2 and GAPDH mRNA levels were determined by RT-qPCR on RIP and input samples. Graph shows relative COX-2 mRNA binding (normalized to GST-TTP wild-type control) as ΔΔCt values (mean ± SD) from four independent experiments (n = 4). Statistical significance was determined by one-way ANOVA followed by Sidak's multiple comparisons test; *p* values are indicated. COX-2, cyclooxygenase-2; HuR, Hu antigen R; PKA, protein kinase A; TTP, tristetraprolin.

**Figure 5:**
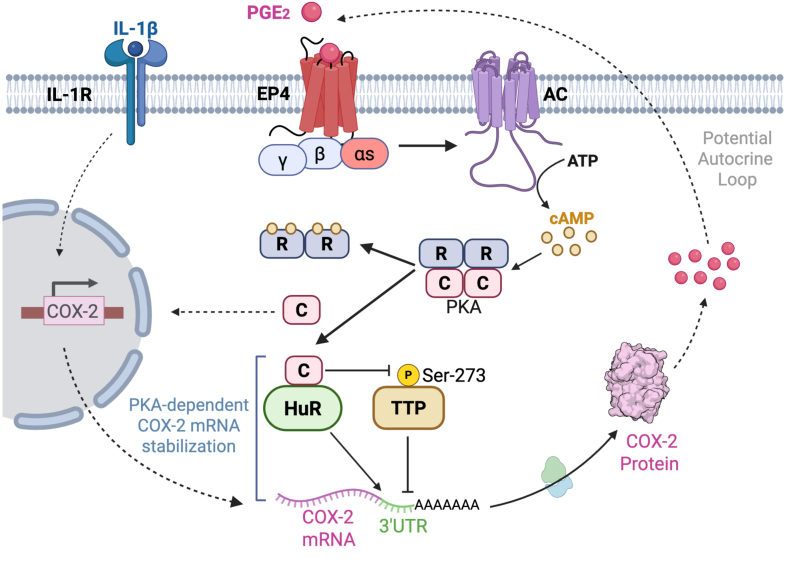
**Proposed model for PKA-dependent posttranscriptional regulation of COX-2 expression *via* HuR and TTP**. Active Gs-coupled EP4 receptors stimulate adenylate cyclase, generating cAMP that binds to PKA regulatory subunits and promotes holoenzyme dissociation. Free PKA catalytic subunits (PKA-C) bind to HuR and phosphorylate TTP at serine 273, inhibiting TTP binding to AU-rich elements in the COX-2 3′UTR. This favors HuR binding and stabilizes COX-2 mRNA. PKA-C binding to HuR provides proximity to COX-2 mRNA, efficiently protecting it from TTP-mediated degradation. Stabilized COX-2 mRNA enhances translation, and the resulting COX-2 protein increases PGE_2_ production. Secreted PGE_2_ can bind autocrinally to EP4 receptors, establishing a potential positive feedback loop. COX-2, cyclooxygenase-2; PKA, protein kinase A; TTP, tristetraprolin.

We hypothesized that, in addition to the interaction of PKA with HuR, COX-2 stabilization might involve the phosphorylation of HuR or TTP. To investigate this, we performed pull-downs from HEK293T cells expressing GST-tagged HuR or TTP constructs, followed by immunodetection using PKA-phosphosubstrate antibodies (PKAS, RRXX∗S/T). We did not detect a phosphorylation signal in the HuR pull-down. However, we observed a strong phosphorylation signal in the TTP sample (Figs. 4*A* and S4*A*). This phosphorylation was sensitive to PKA inhibition with BLU0588 (Fig. 4*B*). To further confirm these results, we performed pull-downs of the GST-TTP construct from HEK293T cells stimulated with forskolin and IBMX. We found that this stimulation led to a transient increase in the TTP-specific phosphorylation signal (Figs. 4*C* and S4*B*), supporting the idea that TTP is a PKA substrate. We then perform an *in silico* search of potential PKA phosphorylation sites for TTP using the ScanSite4.0 platform and the PhosphoSitePlus database. We identified four potential PKA phosphorylation sites: S197, T246, T257, and S273, all located in the C-terminal domain of TTP (Fig. 4*D*). Through site-directed mutagenesis, followed by GST pull-down and PKA substrate-immunodetection, we found that the S273A mutant completely lost the PKA-phosphosubstrate signal (Fig. 4*E*), suggesting that PKA stimulates the phosphorylation of TTP specifically on S273. We hypothesized that this phosphorylation would negatively affect TTP binding to COX-2 mRNA. To test this, we performed RNA Immunoprecipitation and qPCR experiments using GST-TTP wild-type or S273A from HEK293T cells coexpressing the 3′UTR of COX-2 fused to luciferase. Cells were stimulated with forskolin and IBMX to activate PKA. We found no difference in COX-2 3′UTR binding between TTP wild-type and S273A under basal conditions (Fig. 4*F*). However, forskolin/IBMX treatment decreased COX-2 3′UTR binding to TTP wild-type, but it did not alter binding to TTP S273A (Fig. 4*F*). This suggests that phosphorylation at serine 273, stimulated by PKA, negatively affects TTP binding to COX-2 mRNA, contributing to the PKA-mediated stabilization effect through HuR. Collectively, our findings identified a novel mechanism for regulating COX-2 expression mediated by PKA, which involves the interaction and phosphorylation of RBPs HuR and TTP (Fig. 5).

## Discussion

COX-2 is a key inflammatory enzyme that synthesizes PGH_2_, the rate-limiting precursor for thromboxane and PGs, including proinflammatory PGE_2_ (1). Unlike the constitutively expressed COX-1, COX-2 expression is inducible and tightly regulated posttranscriptionally by RBPs that modulate mRNA stability by interacting with AREs at the 3′ UTR (7, 10, 22, 23). Here, we demonstrate that PKA controls COX-2 expression by regulating HuR and TTP, the primary stabilizer and destabilizer of COX-2 mRNA, respectively. In THP-1 macrophages, we observed that PGE_2_ stimulation increases COX-2 mRNA and protein expression, an effect mimicked by direct cAMP stimulation using forskolin. These findings in our experimental system are aligned with previous reports in airway smooth muscle cells, neutrophils, and macrophages, where PGE_2_ potentiates COX-2 expression induced by proinflammatory cytokines such as IL-1β and tumour necrosis factor alpha, and by bacterial LPS, albeit by a poorly understood mechanism (16, 19, 24). The PGE_2_-mediated amplification of COX-2 expression is primarily dependent on the Gs-coupled EP4 receptor, aligning with previous studies in a variety of cell types (16, 24, 25). Pharmacological inhibition of PKA with BLU0588 significantly attenuated COX-2 expression induced by PGE_2_ and IL-1β combination, highlighting the critical role of the Gs → cAMP → PKA axis in this process. PKA inhibition abolished COX-2 mRNA expression without affecting protein decay in cycloheximide-based stability assays, suggesting a transcriptional and/or posttranscriptional controlling mechanism. We also found that PKA inhibition accelerated COX-2 mRNA decay in actinomycin D-based mRNA stability experiments, suggesting that PKA exerts a significant role in regulating COX-2 mRNA stability.

Several RBPs, including AUF-1, TIA-1, CUGBP2, HuR, and TTP, have been reported to bind to AREs within the 3′UTR of COX-2 (10, 26). Notably, HuR and TTP exhibit opposing functions: TTP binding recruits the CCR4-NOT protein complex (CNOT_1-11_), which 3′-5′ exonuclease activity leads to poly(A) tail shortening and mRNA destabilization, while HuR competitively blocks TTP binding, preventing poly(A) tail deadenylation and stabilizing COX-2 mRNA (9, 21). Utilizing a PKA affinity capture approach with cAMP analog-coupled resins, we identified the interaction of endogenous PKA holoenzyme with HuR, but not with TTP. This specific binding of HuR to the PKA catalytic subunits was confirmed by pull-down and immunoprecipitation assays. In addition, domain mapping experiments demonstrated that RRM2 was the primary HuR domain involved in the interaction with PKA. Interestingly, this interaction is dynamically enhanced by cAMP stimulation. This observation contrasts with canonical PKA interactions *via* A-kinase anchoring proteins (AKAPs), which bind to the regulatory (R) subunits and compartmentalize the holoenzyme, thereby facilitating the spatiotemporal control of PKA signaling (27). The recent concept of C-kinase anchoring proteins (C-KAPs), which directly interact with the PKA catalytic subunits (28), suggests that HuR may have additional functions as a C-KAP, thus potentially regulating PKA localization and substrate access. Ultimately, our finding that PKA interacts directly with HuR opens avenues to explore the large repertoire of human RBPs (≥1900) as potential mediators of cAMP signaling through PKA catalytic subunits (29).

We observed that pharmacological inhibition of HuR significantly diminished COX-2 expression in macrophages induced by a combination of PGE_2_ and IL-1β. This reduction in COX-2 expression correlated with a decrease in PGE_2_ production. Furthermore, cAMP stimulation using forskolin and IBMX increased the binding of HuR to the COX-2 transcript in RNA immunoprecipitation assays, suggesting that PKA activation enhances HuR binding to COX-2 mRNA. These findings align with previous studies demonstrating that HuR inhibition reduces COX-2 mRNA stability and expression in human primary monocytes and colon cancer cells (30, 31). Notably, in inflammatory colitis and intestinal cancer models, PKA stimulation by PGE_2_ can amplify COX-2 expression and PGE_2_ synthesis induced by proinflammatory cytokines, such as tumour necrosis factor alpha and IL-1β, in colorectal tumor-associated neutrophils (16). Taken together, our results provide a suitable mechanism by which PKA can amplify COX-2 expression by stabilizing its mRNA through HuR, thus establishing a link for an autocrine feedback loop that leads to increased COX-2 expression and PGE_2_ synthesis.

We investigated whether PKA directly phosphorylates HuR or TTP. Pull-down assays combined with PKA-phosphosubstrate immunodetection suggested that PKA does not stimulate HuR phosphorylation. Surprisingly, we observed remarkable PKA-stimulated phosphorylation of TTP, the functional antagonist of HuR, which was further enhanced upon cAMP stimulation. We identified serine 273 within the C-terminal domain of TTP as the precise phosphorylation site. Comparing wild-type and nonphosphorylatable TTP mutant, we found that S273 phosphorylation diminished TTP binding to the COX-2 3′UTR. Collectively, these results suggest that PKA regulates COX-2 expression by differentially modulating HuR and TTP, specifically by interacting with and stimulating HuR function, and by inhibiting TTP through phosphorylation at S273. Notably, prior *in vitro* phosphorylation studies demonstrated that murine TTP can be phosphorylated by MK2 at serine 266 (S266), which corresponds to serine 273 (S273) in human TTP, which represents the primary PKA phosphorylation site identified by us (32). In this case, however, S266 was considered a minor phosphorylation site when compared to S52 and S178. MK2, like PKA, exhibits a preference for polybasic motifs, suggesting it may also phosphorylate TTP at S273, albeit to a lesser extent. In this case, MK2 is activated downstream from p38 MAPK stimulation by inflammatory cytokines (32). However, in cellular contexts in which cAMP signaling is dominant, such as upon PGE_2_ stimulation of EP4 receptors, PKA is likely the primary kinase responsible for TTP phosphorylation at this residue. Further investigation may be required to elucidate fully how phosphorylation of TTP at S273 results in reduced association with the COX-2 3′UTR, thereby increasing COX-2 mRNA stability and protein expression.

Overall, our data suggest that PKA modulates the contrasting activities of HuR and TTP on COX-2 mRNA. HuR is implicated in the progression of colorectal and pancreatic cancers (33). Notably, these tumors often harbor activating mutations in the Gαs gene (*GNAS*), resulting in hyperactivation of the cAMP→PKA pathway (9, 34, 35, 36, 37). Therefore, PKA may play a role in regulating HuR and TTP in cancer cells expressing active Gαs mutants, thereby stabilizing oncogenic and proinflammatory transcripts, including COX-2. In turn, we provide evidence that PKA may facilitate autocrine and paracrine signaling within the tumor microenvironment cells, such as tumor-associated macrophages, through PGE_2_ secretion, potentially enhancing the EP4→PKA→HuR/TTP→COX-2 signaling axis (Fig. 5). Specifically, our findings reveal a PKA-dependent mechanism that promotes COX-2 expression by directly connecting this protein kinase to two key COX-2 mRNA stability regulators, HuR and TTP. This study broadens our understanding of how signaling proteins control these RBPs. Specifically, our model proposes that the PKA catalytic subunit promotes COX-2 mRNA stability *via* two events: (1) by directly interacting with HuR and (2) by phosphorylation and inhibition of TTP. Consequently, our findings provide a direct link between PKA activity and COX-2 expression through the direct interaction with these effector RBPs, ultimately expanding the repertoire of known mechanisms that intersect intracellular signal transduction events with posttranscriptional regulatory processes.

## Experimental procedures

### Cell lines

THP-1 (TIB-202) and HEK293T (CRL-3216) cell lines (American Type Culture Collection) were maintained in RPMI-1640 (Gibco 11875093) and Dulbecco's Modified Eagle's Medium (Sigma-Aldrich, D6429), respectively. Both media were supplemented with 10% heat-inactivated fetal bovine serum and 1X Anti-Anti (Gibco 15240062). THP-1 medium was further supplemented with 0.05 mM 2-mercaptoethanol (21–985–023). For macrophage differentiation, THP-1 cells were seeded at 5 x 10^5^ cells/well in 6-well plates and cultured overnight with 50 ng/ml phorbol 12-myristate 13-acetate (PMA).

### Antibodies

Antibodies for COX-2 (#12282), GAPDH (#2118), pVASP S157 (#84519), VASP (#3132), pP38 T180/182 (#4511), P38 (#8690), pNFκB S536 (#3033), NFκB (#8042), TTP (#71632), RIα/β (#3927), Cα (#5842), GFP (#2956), GST (#2622), phospho-CREB S133 (#9198), CREB (#4820), and PKA substrates (#9624) were purchased from Cell Signaling Technology. Antibody for HuR (#39–0600) was purchased from Invitrogen and Flag-M2 (F3165) from Millipore-Sigma. Secondary antibodies for anti-rabbit (#4030–05) and anti-mouse (#1030–05) were purchased from Southern Biotech. A complete list of antibodies is provided in Table S1.

### Plasmids and transfection

GST-tagged HuR and TTP constructs were generated by amplifying human HuR from pCMV3-C-Myc-HuR (Sino Biological, HG17509-CM) and human TTP from pFUW-TetO-ZFP36 (Addgene, 178438), respectively. These sequences were then cloned into pCEFL-GST vector, previously digested with BamHI-HF (NEB, R3136S) and dephosphorylated with rSAP (NEB, M0371S), using Gibson Assembly (NEB, E2621). The vector was purified with a QIAquick PCR Purification Kit (QIAGEN, 28104) prior to insertion. TTP mutants (S197A, T246A, S257A, and S273A) were generated using the QuikChange II mutagenesis kit according to the manufacturer's protocol. For the Halo-tagged HuR construct, human HuR was amplified and cloned into pHTN-HaloTag-CMV-neo-Vector (Promega, G7721) using Gibson Assembly. The vector was previously digested with EcoRI-HF (NEB, R3101S), dephosphorylated, and purified. A complete list of primers used for cloning and mutagenesis is provided in Table S2.

The pMirTarget-3′UTR-PTGS2 (COX-2)-luciferase construct was purchased from OriGene (SC219115). pLVX-PKA-Cα was generated *via* Gateway cloning from a pENTRY vector containing human PKA-Cα. pEGFP-C1-PRKACA and pcDNA3.1-Flag-PKA-RIα were previously described (38, 39). All plasmid identities were confirmed by full sequencing.

For plasmid transfection, HEK293T cells were seeded in poly-D-lysine-coated 6-well plates (3.5 x 10^5^cells/well) or 10cm plates (2 x 10^6^cells) and transfected with the indicated plasmids using TurboFect (Thermo Fisher Scientific, 0531) following the manufacturer's protocol.

### Culture of bone marrow-derived macrophages

Primary mBMDMs were isolated from C57BL/6 mice and cultured in Advanced Dulbecco's Modified Eagle's Medium/F12 medium (Gibco, 12634010) supplemented with 10% heat-inactivated fetal bovine serum, 1X Anti-Anti, and 20 ng/ml recombinant mouse M-CSF (PeproTech 315–02) at 37 °C in a humidified 5% CO2 incubator. Bone marrow cells were cultured for 7 days, with fresh M-CSF added every 2 days. On day 7, cells were detached using trypsin-EDTA (Gibco 25300054) and seeded at 5 x 10^5^ cells/well in 6-well plates for experiments. Animal studies were approved by the University of California San Diego (UCSD) Institutional Animal Care and Use Committee (IACUC) under animal study protocol S15195 and adhered to relevant ethical regulations for animal research.

### Lentivirus production and transduction

pLentiCRISPRv2 constructs containing gRNAs targeting HuR (TTGGGGGGATCATCAACTCG) and PKA-Cα (TGAACCTTCCGATCCGCCGT) were purchased from Genscript. HEK293T cells, seeded in poly-D-lysine-coated 10-cm plates, were cotransfected with pCMV-VSV (envelope vector), psPAX2 (packaging vector), and pLentiCRISPRv2 plasmids using Turbofect reagent. Supernatants were collected at 48 and 72 h posttransfection, and lentivirus was concentrated using Lenti-X concentrator (Takara, 631232). HEK293T cells, seeded in 6-well plates, were transduced with lentiviral particles at a multiplicity of infection of 1:1 and selected with 1 μg/ml puromycin (Gibco, A1113803) for 7 days. HuR and PKA-Cα knockout efficiency was confirmed by western blot.

### Cell stimulation and lysate preparation

THP-1 or bone marrow-derived macrophages (BMDMs) were seeded in 6-well plates (5 x 10^5^ cells/well) and serum-starved for 12 h. Cells were then stimulated with 16,16-dimethyl-PGE_2_ (Cayman, 14750), forskolin (Sigma-Aldrich, F6886), IBMX (Sigma-Aldrich, I5879), or recombinant human or mouse IL-1β (BioLegend, 579406 and 575106) alone or in combination (doses and times are specified in figure legends). For inhibition studies, cells were preincubated with EP4 antagonist ONO AE3-208 (Tocris, 3565), PKA inhibitor BLU0588 (TargetMol, T60169), or HuR inhibitor MS-444 (MCE, HY-100685) during the starvation period. Following stimulation, cells were washed once with cold PBS and lysed with 200 μl of RIPA lysis buffer (Pierce, 89900) supplemented with protease and phosphatase inhibitor cocktail (Thermo Fisher Scientific, 78446) and sodium orthovanadate (NEB, P0758S). Cell extracts were scraped, collected, and centrifuged at 16,000*g* for 10 min at 4 °C. Supernatants were collected, protein concentrations were quantified, samples were prepared with 4X Laemmli buffer (Bio-Rad, 1610747), and heated at 95 °C.

### GST pull-down and immunoprecipitation

HEK293T cells, seeded in 6-well plates, transfected, serum-starved, and stimulated, were lysed with 1 ml of immunoprecipitation (IP) buffer (Pierce, 87788) supplemented with protease and phosphatase inhibitors. Cell extracts were scraped, collected, and centrifuged at 16,000*g* for 10 min at 4 °C. Supernatants were collected and incubated with 25 μl of glutathione Sepharose4B (Sigma-Aldrich, GE17–0756–01) for pull-down experiments or DYKDDDDK Fab-Trap agarose (Proteintech, ffa) for immunoprecipitation, with continuous rotation at 4 °C for 2 h. A 75 μl aliquot of supernatant was saved as input and prepared with 4X Laemmli buffer. Following incubation, agarose beads were washed three times with 1 ml of IP buffer, eluted with 50 μl of 2X Laemmli buffer, and heated at 95 °C for 5 min. Pull-down and input samples were analyzed by immunoblot.

### Western blot

Protein samples prepared with Laemmli buffer were resolved on SDS/10% polyacrylamide electrophoresis gels and transferred to polyvinylidene difluoride membranes (Millipore; IPVH304F0). Membranes were blocked and probed with appropriate primary antibodies overnight at 4 °C. After incubation for 1 h at room temperature with the secondary horseradish peroxidase–conjugated antibodies, reaction products were developed with Pierce ECL Western Blotting Substrate (Thermo Fisher Scientific, 32106) or Immobilon ECL UltraPlus Western HRP Substrate (Millipore; WBULP).

### cAMP pull-down assay

THP-1 cells were seeded in two 10-cm plates (5 x 10^6^ cells per plate), differentiated into macrophages with PMA for 24 h, serum-starved for 12 h, and then stimulated with LPS for 2 h. Cells were washed once with cold PBS and lysed with 1 ml of IP buffer supplemented with protease and phosphatase inhibitor cocktail, sodium orthovanadate, and IBMX. Cell extracts were scraped, collected in 1.5 ml tubes, and centrifuged at 16,000*g* for 10 min at 4 °C. Supernatants were collected, pooled, divided into equal volumes, and incubated with 20 μl of 8-AHA-cAMP- or Rp-8-AHA-cAMPS-agarose (Biolog, A012 and A028) or HaloLink-agarose (Promega, G1914) as a negative binding control, with continuous rotation at 4 °C for 3 h. A 75 μl aliquot of the pooled sample was prepared with 4X Laemmli buffer as input. After incubation, agarose beads were washed three times with IP buffer, eluted with 50 μl of 2X Laemmli buffer, and heated at 95 °C for 5 min. Pull-down and input samples were analyzed by immunoblot.

### Production and purification of HuR and PKA-Cα

HuR was purified from HEK293T cells using the HaloTag method. Four 10-cm plates (approximately 7 x 10^6^ cells per plate) were transfected with HaloTag-HuR, lysed with 1 ml of IP lysis buffer supplemented with protease and phosphatase inhibitors, scraped, briefly sonicated on ice, and centrifuged at 16,000*g* for 10 min at 4 °C. Supernatants were pooled and incubated with 100 μl of HaloLink resin with continuous rotation for 12 h at 4 °C. Beads were washed three times with normal IP buffer, three times with IP buffer containing 300 mM NaCl, and once with normal IP buffer. HuR was eluted from the beads using HaloTEV protease in IP buffer.

For the preparation of immobilized Flag-PKA-Cα and Flag-HaloTag (negative control), four 10-cm plates (approximately 7 x 10^6^ cells per plate) were transfected with Flag-PKA-Cα or Flag-HaloTag, lysed with 1 ml of IP lysis buffer supplemented with protease and phosphatase inhibitors, scraped, briefly sonicated on ice, and centrifuged at 16,000*g* for 10 min at 4 °C. Supernatants were incubated with 100 μl of DYKDDDDK Fab-Trap agarose with continuous rotation for 12 h at 4 °C. Beads were washed three times with 1 ml of normal IP buffer, three times with IP buffer containing 225 mM NaCl, and once with normal IP buffer. The purity of soluble and immobilized proteins was evaluated by Coomassie blue staining and identity confirmed by western blot.

### PKA and HuR *in vitro* interaction assay

Approximately 400 ng of HuR protein was incubated with 25 μl of agarose beads containing immobilized Flag-PKA-Cα or Flag-HaloTag in 350 μl of IP buffer with continuous rotation for 3 h at 4 °C. After incubation, the agarose beads were washed four times with IP buffer, eluted with 50 μl of 2X Laemmli buffer, and heated at 95 °C for 5 min. Pull-down and input samples were analyzed by immunoblot.

### Quantitative PCR

THP-1 macrophages, HEK293T cells, or BMDMs were seeded in 6-well plates (5 x 10^5^ cells/well) and treated as indicated in the figure legends. Total RNA was isolated from cells using the RNeasy Mini Kit (QIAGEN, 74104) according to the manufacturer's instructions. RNA concentration was quantified using a NanoDrop ND-1000 spectrophotometer (Thermo Fisher Scientific). Complementary DNA (cDNA) was synthesized from 0.5 to 2 μg of RNA using SuperScript VILO Master Mix (Invitrogen, 11755050) following the manufacturer's protocol. qPCR was performed using Fast SYBR Green Master Mix (Applied Biosystems, 4385612) on a CFX Opus 384 Real-Time PCR system (Bio-Rad) to analyze human and murine COX-2, GAPDH, and PTGER1-4 receptor expression. Primer sequences for qPCR are listed in Table S3.

### RNA immunoprecipitation (RIP) assay

HEK293T cells, seeded in 10-cm plates, were transfected with GFP-HuR or GST-TTP constructs. Cells were serum-starved for 12 h and then stimulated with forskolin and IBMX for 1 h. Cells were lysed with IP buffer supplemented with protease, phosphatase, and RNase inhibitors (Invitrogen, AM2694). Cell extracts were scraped from the plates, collected, briefly sonicated, and centrifuged at 16,000*g* for 10 min at 4 °C. Supernatants were incubated with 50 μl of GFP-Trap Agarose (Proteintech, gta) or Glutathione Sepharose with continuous rotation for 12 h at 4 °C. A 50 μl aliquot of the supernatant was saved as input and prepared with 300 μl of RLT buffer (QIAGEN). Beads were washed five times with 1 ml of IP buffer, and proteins and RNA were eluted from the beads with 350 μl of RLT buffer. RNA was extracted using the RNeasy kit and quantified using a NanoDrop spectrophotometer. Equal amounts of RNA were used to synthesize cDNA by RT-PCR, which was then used for qPCR analysis of COX-2 and GAPDH. RIP ΔΔCt values were calculated and normalized to input values.

### mRNA decay assays

THP-1 macrophages were serum-starved for 12 h, stimulated with PGE_2_ and IL-1β for 2 h, followed by the addition of 5 μg/ml actinomycin D to inhibit transcription for the indicated time points, in the presence or absence of 1 μM PKA inhibitor BLU0588. Total RNA was isolated, cDNA was synthesized, and transcript abundance was determined by qPCR.

### Protein decay assays

THP-1 macrophages were serum-starved for 12 h, stimulated with PGE_2_ and IL-1β for 6 h, followed by the addition of 1 μM cycloheximide to inhibit translation for the indicated time points, in the presence or absence of 1 μM PKA inhibitor BLU0588. Cells were lysed with 200 μl of RIPA lysis buffer supplemented with protease and phosphatase inhibitor cocktail and sodium orthovanadate. Cell extracts were scraped, collected, and centrifuged at 16,000*g* for 10 min at 4 °C. Supernatants were collected, protein concentrations were quantified, samples were prepared with 4X Laemmli buffer, and heated at 95 °C. COX-2 protein abundance was determined by western blot.

### PGE_2_ measurement

THP-1 cells were seeded in 6-well plates (1 x 10^6^ cells/well), differentiated into macrophages, serum-starved, and incubated with dimethyl sulfoxide or 10 μM HuR inhibitor MS-444 during the starvation period. The starvation medium was then refreshed, and cells were stimulated with a combination of forskolin, IBMX, and IL-1β for 6 h. Supernatants were collected and used for ELISA-based PGE_2_ measurements (Cayman, 514010) according to the manufacturer's instructions.

### Generation of structural models by AlphaFold 3

Structural models of the PKA-Cα/HuR complex were generated using the AlphaFold 3 server (40). The inputs for modeling, including amino-acid sequences, PTMs, ligands, and ions are detailed in Table S4. To ensure an accurate modeling of PKA-Cα active state, we included as inputs two Mg^2+^ ions, one molecule of ATP and the phosphorylation of residues T198 and S339, which are relevant PTMs for the kinase activity. Twenty-five models were generated for PKA-Cα in complex with a small substrate peptide (from TTP) or with the RRM1-RRM2 domains of HuR. ipTM Scores from the corresponding models are displayed as violin plots in Figure S2*E*. Structures were visualized and analyzed in ICM v3.9-3a (Molsoft LLC). The models with the highest ipTM score were selected as the representative structures for Figure S2, *B*, *C* and *D*. PKA regulatory subunit binding site in PKA-Cα (residues 191–197, VKGRTWT) was previously reported (41, 42). The RNA binding sites of HuR RRM1 and RRM2 (K50, F65, R136, and F151) located at the four-stranded β-sheet were inferred from the structure of HuR RRM3 in complex with 5′UUUUUU3′ RNA (PDB ID: 6G2K) (43) *via* alignments of the primary sequences of HuR RRM1, RRM2, and RRM3.

### Statistical analysis

All values are represented as mean ± SD. Data from at least three independent experiments were analyzed, and statistics performed using GraphPad Prism software using Student’s unpaired two-tailed *t* test (for comparing two groups) and ANOVA (for comparing three or more groups). *p* Values of less than 0.05 were considered significant.

All the illustrations for this manuscript were prepared using Biorender.

## Data availability

All data associated with this study are presented within the article.

## Supporting information

This article contains supporting information.

## Conflict of interest

J. S. G. reports consulting fees from Radionetics Oncology, BTB Therapeutics, and Acurion, and is the founder of Kadima Pharmaceuticals, all of which are unrelated to the current study. The other authors declare that they have no conflicts of interest with the contents of this article.
