# Supplementary material for: Protein kinase a regulates cyclooxygenase-2 expression through the RNA-binding proteins HuR and TTP
Source: J Biol Chem. 2025 Dec 18;302(2):111064. doi: 10.1016/j.jbc.2025.111064 (PMC12914655; doi:10.1016/j.jbc.2025.111064)

Supplemental Figure 1. Additional experiments related to prostaglandin receptors, COX-2 stability, and PGE_2 secretion.

A, Prostaglandin receptor (PTGER) expression in THP-1 macrophages (Mφ). Cells were serum-starved for 12 hours, lysed, and subjected to RNA extraction, RT-PCR, and qPCR for PTGER1-4 and GAPDH. Graph shows ΔΔCt values (mean ± SD) from three independent experiments (n=3). Statistical significance was determined by one-way ANOVA followed by Sidak's multiple comparisons test; p values are indicated. *B*, Effect of PKA on COX-2 protein stability in THP-1 MΦ. Cells were serum-starved for 12 hours, stimulated with 1 μM PGE_2 and 5 ng/mL IL-1β for 6 hours, and then incubated with 1 μM cycloheximide for the indicated times in the presence of 1 μM BLU0588 (PKA inhibitor) or DMSO (control). Cell lysates were analyzed by immunoblotting for COX-2 and GAPDH. Representative blots from three independent experiments are shown.

Supplementary Figure 1. Additional experiments related to Prostaglandin receptors expression and COX-2 protein stability.

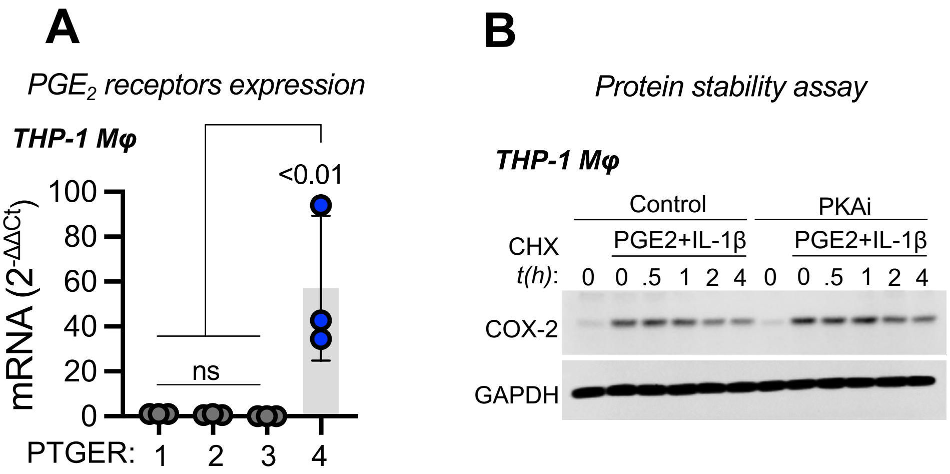

Supplement: Figure S1 [file mmc1.pdf]
