# Supplementary material for: Protein kinase a regulates cyclooxygenase-2 expression through the RNA-binding proteins HuR and TTP
Source: J Biol Chem. 2025 Dec 18;302(2):111064. doi: 10.1016/j.jbc.2025.111064 (PMC12914655; doi:10.1016/j.jbc.2025.111064)

Supplemental Figure 2. Additional information regarding the PKA/HuR interaction and PGE2 secretion.

A, Diagram illustrating the use of cAMP analog agarose beads to differentially isolate PKA complexes. (i) Regulatory subunit isolation: Immobilized 8-AHA-cAMP (agonist) binds to the PKA regulatory (R) subunits, causing a conformational change that promotes the dissociation of the catalytic (C) subunit. This results in the specific isolation of the regulatory subunits. (ii) Holoenzyme isolation: Immobilized Rp-8-AHA-cAMPS (an antagonist) binds to the regulatory subunits but prevents the binding of endogenous cAMP and blocks the dissociation of the catalytic subunit, thus preserving the PKA holoenzyme during isolation. *B*, A representative structural model of PKA catalytic subunit alpha, generated using AlphaFold 3.0. Key functional regions are highlighted by colored spheres: Blue spheres highlight the major binding interface for the regulatory subunit. Dark red spheres mark the catalytic and ATP binding sites. A small substrate peptide from TTP (yellow strand) was introduced into the model as an additional strategy to improve the visualization of the catalytic pocket in our model *C*, A representative structural model of the RNA Recognition Motifs 1 and 2 (RRM1 and RRM2 domains) of HuR, generated using AlphaFold 3.0. The magenta spheres highlight key conserved residues within the RRM domains that are involved in the binding of target RNA molecules *D*, A representative structural model of the PKA-Cα and HuR (RRM1-RRM2) complex. This model predicts that the interaction interface is predominantly formed between PKA-Cα and the second RRM domain of HuR. The regulatory subunit binding surface and catalytic site in PKA-Cα and RNA-binding sites of RRM1 remain accesible in this complex. *E*, Violin plot of the ipTM values from 25 independent models obtained for the PKA-Cα and PKA-Cα/HuR complex. *F*, Effect of HuR inhibition on PGE_2 secretion. THP-1 Mφ were serum-starved in the presence or absence of 10 µM MS-444 (HuR inhibitor) for 12 hours and then stimulated with 1 µM forskolin, 50 µM IBMX and 5 ng/mL IL-1β for 6 hours. Supernatants were collected, and PGE_2 concentrations were measured by ELISA. Graph shows fold-change in PGE_2 concentration from three independent replicates. Statistical significance was determined by one-way ANOVA followed by Sidak's multiple comparisons test; *p* values are indicated.

Supplementary Figure 2. Additional information regarding the PKA/HuR interaction and PGE2 secretion.

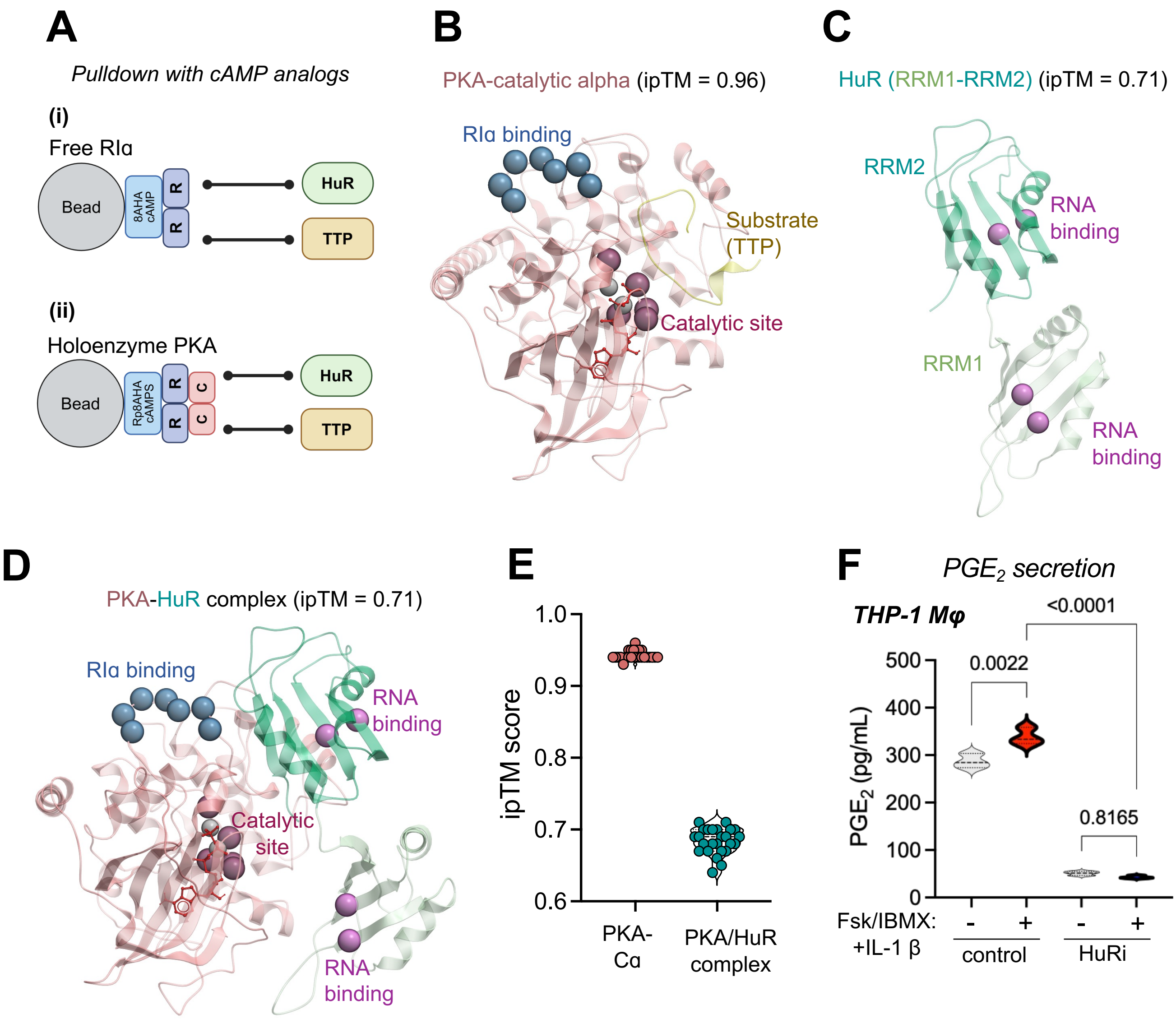

Supplement: Figure S2 [file mmc2.pdf]
