# Supplementary material for: Protein kinase a regulates cyclooxygenase-2 expression through the RNA-binding proteins HuR and TTP
Source: J Biol Chem. 2025 Dec 18;302(2):111064. doi: 10.1016/j.jbc.2025.111064 (PMC12914655; doi:10.1016/j.jbc.2025.111064)

Supplemental Figure 3. Additional experiments related to PKA and HuR knockout effects and HuR binding.

A, Effect of PKA-Cα knockout (KO) on COX-2 expression induced by forskolin and IBMX stimulation. HEK293T cells (Cas9 control or PKA-Cα KO) were serum-starved for 12 hours and stimulated with 1 μM forskolin and 50 μM IBMX for the indicated times. Cell lysates were analyzed by immunoblotting for COX-2, phospho-PKA substrates, PKA-Cα, HuR, and GAPDH. Representative blots from three independent experiments are shown.

B, Effect of HuR knockout (KO) on COX-2 expression induced by forskolin and IBMX stimulation. HEK293T cells (Cas9 control or HuR KO) were treated as in *A*. Cell lysates were analyzed by immunoblotting for COX-2, phospho-PKA substrates, PKA-Cα, HuR, and GAPDH. Representative blots from three independent experiments are shown. *C*, Effect of HuR knockout (KO) on CREB phosphorylation. HEK293T cells (Cas9 control or HuR KO) were serum-starved for 12 hours and stimulated with 1 μM forskolin and 50 μM IBMX for 2 hours. Cell lysates were analyzed by immunoblotting for phospho-CREB S133, phospho-PKA substrates, HuR, and GAPDH. Representative blots from three independent experiments are shown. *D*, Effect of HuR knockout (KO) on cAMP-response element (CRE) activity. HEK293T cells expressing pGL4.29-luc2P/CRE/Hygro and pCEFL-Flag-renilla-luc were serum-starved for 12 hours and stimulated with 1 μM forskolin and 50 μM IBMX for 2 hours. Cell extracts were prepared for luminescence detection using the Dual-Glo Luciferase Assay System (Promega, E2920) according to the manufacturer's instructions. Graph shows firefly/renilla luminescence ratios (normalized to forskolin/IBMX control) (mean ± SD) from three independent experiments (n=3). Statistical significance was determined by one-way ANOVA followed by Sidak's multiple comparisons test; p values are indicated. *E*, Effect of PKA activation on HuR binding to COX-2 mRNA. HEK293T cells expressing EGFP-HuR or control EGFP were serum-starved, treated with 1 μM forskolin and 50 μM IBMX for the indicated times, lysed, and subjected to RNA immunoprecipitation (RIP). COX-2 and GAPDH amplicons were detected by endpoint PCR (20 cycles) on RIP and input samples. Agarose gels show COX-2 amplicons. Immunoblots show EGFP immunoprecipitation and CREB phosphorylation as stimulation controls.

Supplementary Figure 3. Additional experiments related to PKA and HuR knockout effects and HuR binding.

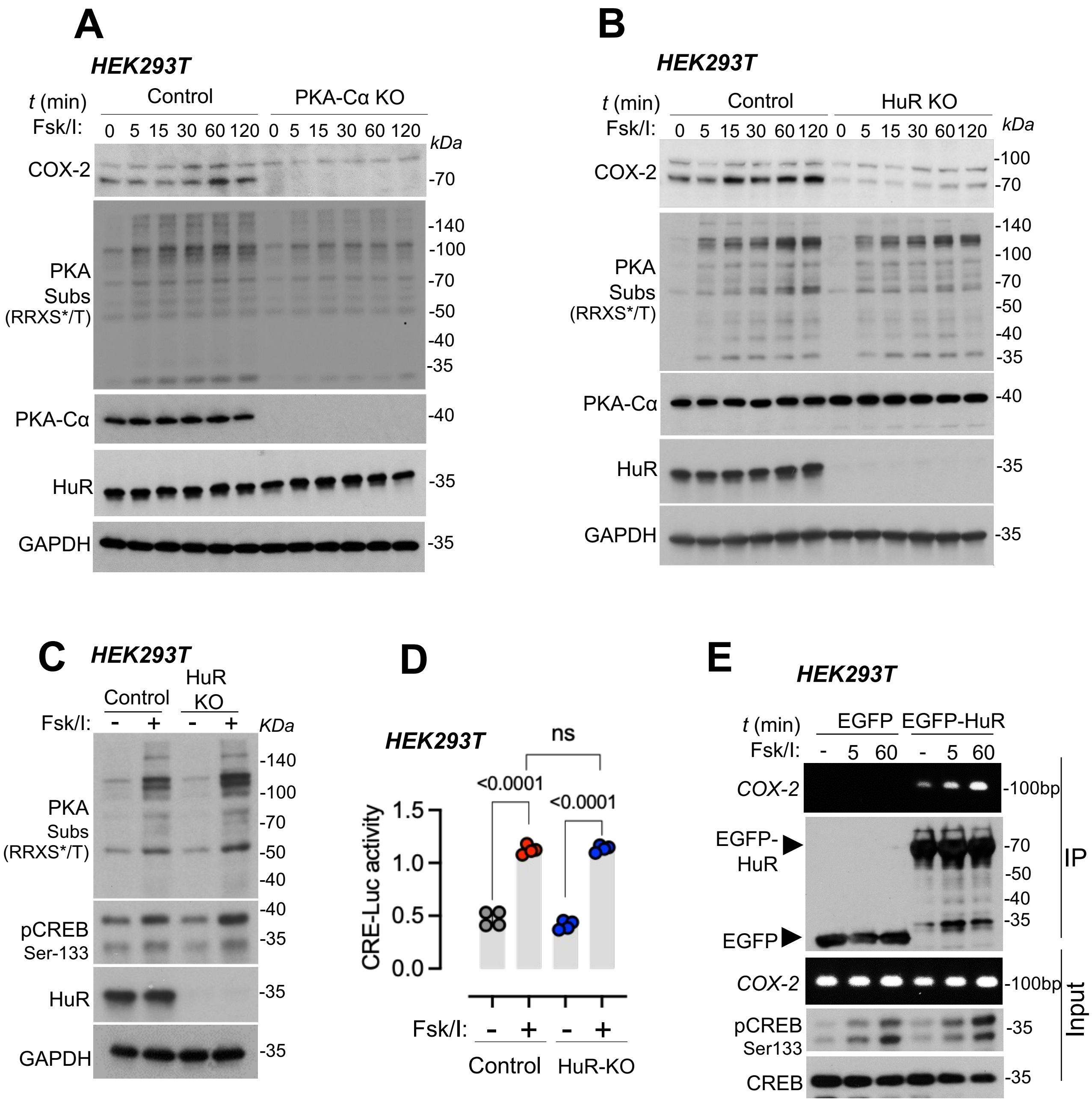

Supplement: Figure S3 [file mmc3.pdf]
