# Supplementary material for: Protein kinase a regulates cyclooxygenase-2 expression through the RNA-binding proteins HuR and TTP
Source: J Biol Chem. 2025 Dec 18;302(2):111064. doi: 10.1016/j.jbc.2025.111064 (PMC12914655; doi:10.1016/j.jbc.2025.111064)

Supplemental Figure 4. Additional experiments related to TTP phosphorylation.
A, Exposure of 4 minutes for PKAS (RRXS*/T*) signal corresponding to Fig. 4*A*. *B*, Quantification of phospho-TTP (PKAS) signal corresponding to Fig. 2*C*. Densitometry of the bands corresponding to phospho-TTP (PKAS) and GST-TTP from pulldown samples was performed. Phospho-TTP (PKAS) intensity values were adjusted to the corresponding GST values and then normalized to control conditions. Graph shows values (mean ± SD) from four independent experiments (n=4). Statistical significance was determined by one-way ANOVA followed by Sidak's multiple comparisons test; *p* values are indicated.

Supplementary Figure 4. Additional experiments related to TTP phosphorylation.

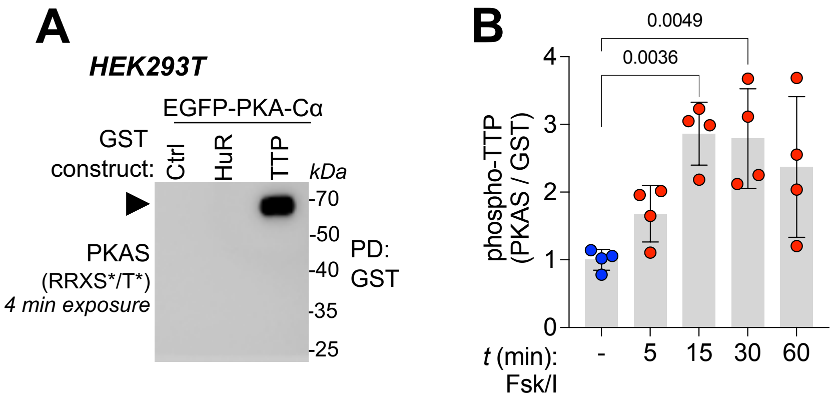

Supplement: Figure S4 [file mmc4.pdf]
