# Supplementary material for: Protein kinase a regulates cyclooxygenase-2 expression through the RNA-binding proteins HuR and TTP
Source: J Biol Chem. 2025 Dec 18;302(2):111064. doi: 10.1016/j.jbc.2025.111064 (PMC12914655; doi:10.1016/j.jbc.2025.111064)
Supplement: Supplementary Tables [file mmc5.docx]

Supplementary Table 1. Antibodies.

| **Antibody** | **Source** | **Identifier** |
| --- | --- | --- |
| COX-2 | Cell Signaling Technology | 12282 |
| GAPDH | Cell Signaling Technology | 2118 |
| Phospho-VASP S157 | Cell Signaling Technology | 84519 |
| VASP | Cell Signaling Technology | 3132 |
| Phospho-P38^MAPK^ T180/182 | Cell Signaling Technology | 4511 |
| P38^MAPK^ | Cell Signaling Technology | 8690 |
| Phospho-NFκB P65 subunit S536 | Cell Signaling Technology | 3033 |
| NFκB P65 subunit | Cell Signaling Technology | 8042 |
| TTP | Cell Signaling Technology | 71632 |
| PKA-R1α/β | Cell Signaling Technology | 3927 |
| PKA-Cα | Cell Signaling Technology | 5842 |
| GFP | Cell Signaling Technology | 2956 |
| GST | Cell Signaling Technology | 2622 |
| phospho-CREB S133 | Cell Signaling Technology | 9198 |
| CREB | Cell Signaling Technology | 4820 |
| Phospho-PKA substrate (RRXS*/T*) | Cell Signaling Technology | 9624 |
| HuR | Invitrogen | 39-0600 |
| Flag-M2 | Millipore-Sigma | F3165 |
| anti-rabbit | Southern Biotech | 4030-05 |
| anti-mouse | Southern Biotech | 1030-05 |

Supplementary Table 2. Primers for cloning.

| **Identifier** | **Sequence** |
| --- | --- |
| GST-HuR forward | CTGGTTCCGCGTGGATCCATGTCTAATGGTTATGAA |
| GST-HuR reverse | GACTACGAATTCAGGGGATCCTTATTTGTGGGACTTGTTGGT |
| GST-TTP forward | GATCTGGTTCCGCGTGGATCCATGGATCTGACTGCCATCTAC |
| GST-TTP reverse | GACTACGAATTCAGGGGATCCTCACTCAGAAACAGAGATGCG |
| Halo-HuR forward | AACGCGATCGCTTCCGAATTCATGTCTAATGGTTATGAAGAC |
| Halo-HuR reverse | CCGCGGTTGAGCTCTGAATTCTTATTTGTGGGACTTGTTGGT |

Supplementary Table 3. Primers for qPCR.

| **Identifier** | **Sequence** |
| --- | --- |
| Human COX-2 forward | CTGGCGCTCAGCCATACAG |
| Human COX-2 reverse | CGCACTTATACTGGTCAAATCCC |
| Human GAPDH forward | CTGGGCTACACTGAGCACC |
| Human GAPDH reverse | AAGTGGTCGTTGAGGGCAATG |
| Human EP1 receptor forward | AGCTTGTCGGTATCATGGTGG |
| Human EP1 receptor reverse | AGCAAGTGTATGACCCTGGTAAT |
| Human EP2 receptor forward | CGATGCTCATGCTCTTCGC |
| Human EP2 receptor reverse | GGGAGACTGCATAGATGACAGG |
| Human EP3 receptor forward | CGCCTCAACCACTCCTACAC |
| Human EP3 receptor reverse | GACACCGATCCGCAATCCTC |
| Human EP4 receptor forward | CCGGCGGTGATGTTCATCTT |
| Human EP4 receptor reverse | CCCACATACCAGCGTGTAGAA |
| Mouse COX-2 forward | TGAGCAACTATTCCAAACCAGC |
| Mouse COX-2 reverse | GCACGTAGTCTTCGATCACTATC |
| Mouse GAPDH forward | AGGTCGGTGTGAACGGATTTG |
| Mouse GAPDH reverse | GGGGTCGTTGATGGCAACA |
| Firefly Luciferase forward | GTGGTGTGCAGCGAGAATAG |
| Firefly Luciferase reverse | CGCTCGTTGTAGATGTCGTTA |

Supplementary Table 4. Inputs for structural modelling using AlphaFold 3. Phosphorylated residues are marked as bold and underlined characters.

| **Name** | **Uniprot ID** | **Sequence** |
| --- | --- | --- |
| ELAVL1/HuR (RRM1-RRM2) | P17612 | NLIVNYLPQNMTQDELRSLFSSIGEVESAKLIRDKVAGHSLGYGFVNYVTAKDAERAINTLNGLRLQSKTIKVSYARPSSEVIKDANLYISGLPRTMTQKDVEDMFSRFGRIINSRVLVDQTTGLSRGVAFIRFDKRSEAEEAITSFNGHKPPGSSEPITVKFAAN |
| PRKACA | Q15717 | MGNAAAAKKGSEQESVKEFLAKAKEDFLKKWESPAQNTAHLDQFERIKTLGTGSFGRVMLVKHKETGNHYAMKILDKQKVVKLKQIEHTLNEKRILQAVNFPFLVKLEFSFKDNSNLYMVMEYVPGGEMFSHLRRIGRFSEPHARFYAAQIVLTFEYLHSLDLIYRDLKPENLLIDQQGYIQVTDFGFAKRVKGRTW**T**LCGTPEYLAPEIILSKGYNKAVDWWALGVLIYEMAAGYPPFFADQPIQIYEKIVSGKVRFPSHFSSDLKDLLRNLLQVDLTKRFGNLKNGVNDIKNHKWFATTDWIAIYQRKVEAPFIPKFKGPGDTSNFDDYEEEEIRV**S**INEKCGKEFSEF |
| ZFP36/TTP  (265-280) | P26651 | LGGLVRTPSVQSLGSD |
| Others |  | 2 Mg^2+^ (ion), 1 ATP (Ligand) |
